# Dietary Supplement of Large Yellow Tea Ameliorates Metabolic Syndrome and Attenuates Hepatic Steatosis in db/db Mice

**DOI:** 10.3390/nu10010075

**Published:** 2018-01-12

**Authors:** Yun Teng, Daxiang Li, Ponmari Guruvaiah, Na Xu, Zhongwen Xie

**Affiliations:** State Key Laboratory of Tea Plant Biology and Utilization, School of Tea and Food Sciences and Technology, Anhui Agricultural University, Hefei 230036, China; tengyunyy@sina.cn (Y.T.); dxli@ahau.edu.cn (D.L.); Ponmariguruvaiah@yahoo.com (P.G.); naxu2014@ahau.edu.cn (N.X.)

**Keywords:** large yellow tea, db/db mice, hepatic steatosis, metabolic syndrome, mechanism

## Abstract

Yellow tea has been widely recognized for its health benefits. However, its effects and mechanism are largely unknown. The current study investigated the mechanism of dietary supplements of large yellow tea and its effects on metabolic syndrome and the hepatic steatosis in male db/db mice. Our data showed that dietary supplements of large yellow tea and water extract significantly reduced water intake and food consumption, lowered the serum total and low-density lipoprotein cholesterol and triglyceride levels, and significantly reduced blood glucose level and increased glucose tolerance in db/db mice when compared to untreated db/db mice. In addition, the dietary supplement of large yellow tea prevented the fatty liver formation and restored the normal hepatic structure of db/db mice. Furthermore, the dietary supplement of large yellow tea obviously reduced the lipid synthesis related to gene fatty acid synthase, the sterol regulatory element-binding transcription factor 1 and acetyl-CoA carboxylase α, as well as fatty acid synthase and sterol response element-binding protein 1 expression, while the lipid catabolic genes were not altered in the liver of db/db mice. This study substantiated that the dietary supplement of large yellow tea has potential as a food additive for ameliorating type 2 diabetes-associated symptoms.

## 1. Introduction

Metabolic syndrome is a group of the most dangerous risk factors associated with type 2 diabetes and cardiovascular disease (CVD) [1,2,3,4,5,6,7,8,9]. According to the joint statement from the International Diabetes Federation (IDF), the American Heart Association (AHA)/National Heart, the Lung and Blood Institute (NHLBI), the World Heart Federation, the International Atherosclerosis Society, and the International Association for the Study of Obesity, a person defined as having metabolic syndrome must have three or more of the following risk factors: obesity, high plasma TG level (≥1.7 mmol/L), high blood pressure (systolic blood pressure ≥ 130 mmHg or diastolic blood pressure ≥ 85 mmHg), high fasting plasma glucose (≥5.6 mmol/L), and reduced HDL cholesterol (<1.0 mmol/L in males, <1.3 mmol/L in females) [3,10,11]. Right now, there are approximately a quarter of adults suffering from metabolic syndrome, and obesity is the primary and direct cause of metabolic syndrome and type 2 diabetes [11,12]. Type 2 diabetes (T2D) is a long-term metabolic disorder that is characterized by high blood glucose and insulin resistance. Diabetic complications include retinopathy, hypertension, cardiovascular diseases, kidney failure, and even ketoacidosis [5]. The db/db mouse is a leptin receptor mutant mouse model that is widely used for the study of T2D and metabolic syndrome [13]. The phenotypes and pathogenesis of T2D in db/db mice are similar to those of human T2D mellitus [13,14]. The mutation mice show hyperphagia due to defects in leptin signaling, leading to obesity, hyperinsulinemia, insulin resistance, dyslipidemia, hyperglycemia, and inflammation at the age of 3–4 months [13,14,15].

Based on various manufacturing processes and the degrees of fermentation, tea—one of the three most popular beverages worldwide—is classified into six main categories: non-fermented green tea, slightly-fermented white tea, partly-fermented yellow tea, semi-fermented oolong tea, fully-fermented black tea, and post-fermented dark tea [16]. At present, in addition to exploring effective drug therapy, increasing attention has been paid to the use of tea as a supplementary treatment for metabolic syndrome [17,18,19,20]. Some reports have suggested that the green tea extract can significantly reduce body weight gain, decrease body mass index (BMI) and levels of total cholesterol (TC), triglyceride (TG), and low-density lipoprotein (LDL) cholesterol, and increase energy expenditure and satiety at mealtime on obese patients [18,19]. Moreover, Kang et al. [20] found that fermented green tea, compared with green tea, had better anti-obesity, hypoglycemic, hypolipidemic, and antioxidant effects in db/db mice and made the mice more resistant to type 2 diabetes. The researchers also focused on the regulation and effect of glucose and lipid metabolism with an epigallocatechin-3-gallate (EGCG), a secondary metabolite of tea. Ortsäter et al. [21] reported that, in db/db mice, EGCG reduced the pathological changes of islets and increased the number and size of islet cells, thus accordingly improving the function of pancreatic secretion, which helped to delay the course of metabolic syndrome. However, it has been proven that high doses of EGCG produced cytotoxicity and hepatotoxicity, which indicates that crude extracts might be safer than a single substance [22,23,24,25].

Large yellow tea, made from “one bud 6 leaves” of tea plant (*Camellia sinensis)*, is one of the most famous traditional yellow teas in China, which is very popular because of its unique burnt flavor. A recent study from the Institute of Cancer Research (ICR) proved that yellow tea had a significant anti-hyperglycemic effect in high-fat-diet-induced mice when compared with green tea, black tea, and dark tea, which confirmed that yellow tea had a certain role in the recovery of glucose metabolism disorder [22]. However, high-fat-diet-induced ICR mice are not typical diabetic mice, and there is a lack of long-term experiments investigating the effect of yellow tea on rodent models of metabolic syndrome. Therefore, the current study comparatively investigated the molecular mechanism of dietary supplements of large yellow tea powder and its crude water extracts and their effects on glucose and lipid metabolism as well as the hepatic steatosis of middle-age db/db mice over a 10 week treatment period.

## 2. Results

### 2.1. Quantitative Analysis of Characteristic Components in Large Yellow Tea and Its Water Extract by High Pressure Liquid Chromatography LYT and LWE by HPLC

The chromatographic pattern obtained from quantitative high-pressure liquid chromatography (HPLC) analysis of large yellow tea and its water extract were compared with standards representing epigallocatechin (EGC), catechin (C), epicatechin (EC), (−)-epigallocatechin-3-gallate (EGCG), (−)-gallocatechin-3-gallate (GCG), (−)-epicatechin-3-gallate (ECG), theanine, and caffeine. The results are listed in Table 1.

The quantitative analysis showed that ECGC was a major constituent identified in both LYT and LWT. The concentration of EGCG in LYT and LWT was 4.250 ± 0.023 mg/g and 13.774 ± 0.543 mg/g, respectively, which produced an almost equal concentration of EGCG in both the 5% large yellow tea and 1.5% water extract diets used to feed db/db mice. Along with higher levels of caffeine and theanine, LWE saw a remarkable amount of total catechins when compared with that in LYT (27.910 ± 0.011 vs. 11.531 ± 0.035 mg/g).

### 2.2. Effect of LYT and LWE on Diabetic Syndrome on db/db Mice

**Figure 1:**
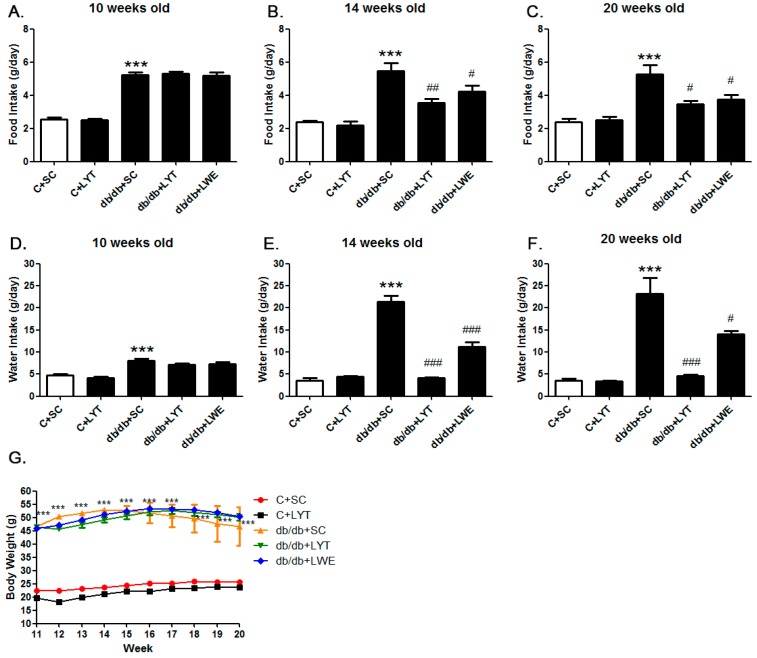
Food consumption (**A**–**C**) water intake (**D**–**F**) and body weight (**G**) of the control and db/db mice. C + SC: control mice fed the standard AIN93 diet (SC); C + LYT: control mice fed SC containing 5% of the large yellow tea powder (LYT); db/db + SC: db/db mice fed SC diet; db/db + LYT: db/db mice fed LYT diet; db/db + LWE: db/db mice fed LWE diet. Values are means ± SE (*n* = 3). *** *p* < 0.001 when compared with the C + SC group; # *p* < 0.05; ## *p* < 0.01; ### *p* < 0.001 when compared with the db/db + SC group.

T2D mellitus is characterized by increased eating and drinking as well as late-stage weight loss [5,12,13,14]. We measured food consumption and water intake of control and db/db mice with different dietary treatments in order to figure out the effect of large yellow tea and its water extract on phenotypes in db/db mice. The food consumption and water intake in the db/db mice were significantly increased when compared with the control mice at 10 weeks old (Figure 1A,D). After four weeks of tea dietary supplement (14 weeks old), db/db + LYT, and db/db + LWE group mice showed a dramatic decrease in both food consumption and water intake when compared with db/db + SC mice (Figure 1B,E). Furthermore, this decrease in food consumption and water intake was persistently observed throughout the experimental period (20 weeks old) (Figure 1C,F). However, there was no difference in food consumption and water intake between the C + SC group and C + LYT group mice (Figure 1A–F). Interestingly, we found that the db/db mice fed LYT had a more profound effect on the reduction of water intake than that of db/db mice fed with the LWE diet (Figure 1E,F).

Moreover, we observed the body weights of the control and db/db mice 10–20 weeks old fed various diets (Figure 1G). In the control groups, the body weights of the mice fed an AIN 93 standard diet (C + SC) or an AIN 93 diet containing 5% large yellow tea powder (C + LYT) were increased slightly from 11 to 20 weeks. There was no difference in body weight gain between the two groups. However, the db/db mice fed with SC showed remarkable obesity, and the body weights were significantly higher than the body weights of the control mice throughout the experimental period (*p* < 0.001). The T2D mouse model has phenotypes of obesity and emaciation in later stages due to diabetic complications [14,26]. In our study, the body weights of db/db mice fed an AIN 93 diet (db/db + SC) gradually increased from 11 to 14 weeks old and then consistently decreased from 14 to 20 weeks old due to the development of diabetic complications. However, the body weights of both the db/db + LWE group and the db/db + LYT group increased gradually from 10 to 16 weeks old and then decreased slowly until 20 weeks old (Figure 1G).

### 2.3. Protective Effect of LYT and LWE on Serum Lipids Profile of db/db Mice

**Figure 2:**
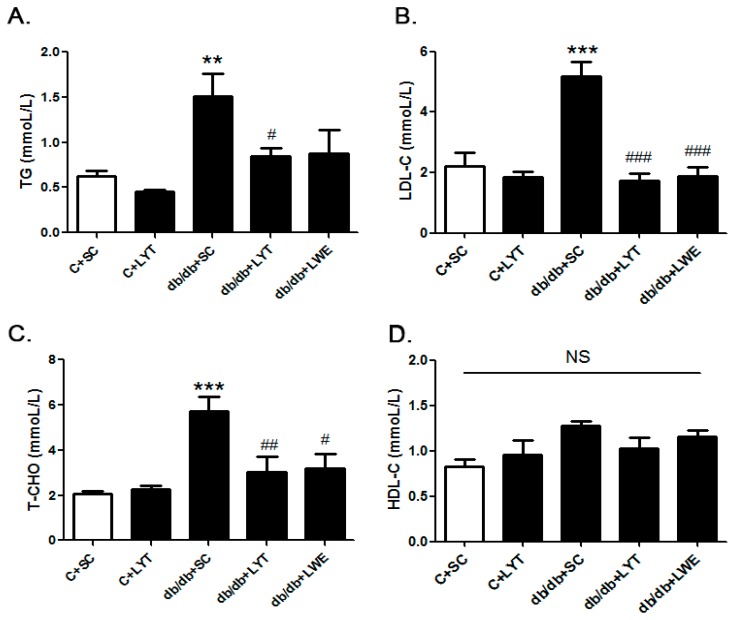
Serum lipid profile of six groups of mice at 20 weeks old. Triglyceride (**A**); low-density lipoprotein cholesterol (**B**); total cholesterol (**C**); and high-density lipoprotein cholesterol (**D**). Values are means ± SE (*n* = 4–6). ** *p* < 0.01; *** *p* < 0.001 compared with C + SC group; # *p* < 0.05; ## *p* < 0.01; ### *p* < 0.001 when compared with the db/db + SC group; NS, no significant difference.

Our data showed that the level of TG, LDL-C, and TC was significantly increased in db/db + SC mice than those in the control mice fed a chow diet (Figure 2A–C). The db/db mice fed a diet containing LYT and LWE showed greater reductions in the level of TG, LDL-C, and TC when compared with that of the db/db mice fed a standard chow diet. Interestingly, after 10 weeks of dietary supplement, the levels of TG, LDL-C, and TC in the db/db + LYT and db/db + LWE group mice were close to the levels in the control mice fed a standard chow diet. However, the tea dietary supplement did not alter the concentration of HDL-C (Figure 2D).

### 2.4. LYT Reduced the Blood Glucose and Improved Glucose Tolerance in db/db Mice

**Figure 3:**
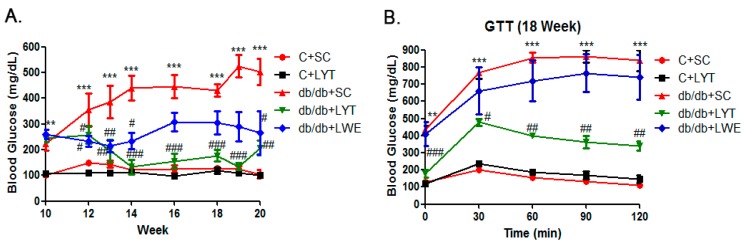
Blood glucose (**A**) and glucose tolerance test (**B**) in five group of mice. Values are means ± SE (*n* = 4–6). ** *p* < 0.01; *** *p* < 0.001 when compared with the C + SC group; # *p* < 0.05; ## *p* < 0.01; ### *p* < 0.001 when compared with the db/db + SC group.

Hyperglycemia and glucose intolerance are major indicators of T2D mellitus [5,13,27]. Accordingly, we dynamically measured blood glucose during the entire experimental period. The data showed that fasting blood glucose levels were significantly elevated in db/db mice fed a standard chow diet compared to the control mice from 10 to 20 weeks old. In addition, the db/db mice were prone to becoming diabetic at the age of 11 weeks, with glucose levels over 300 mg/dL (Figure 3A). Additionally, the db/db mice fed a standard chow diet showed gradually increased blood glucose levels from 300 to approximately 500 mg/dL from 11 to 20 weeks old. Surprisingly, the blood glucose level of db/db mice fed with LYT for 2 weeks was significantly decreased. After four weeks of dietary supplement with LYT, the blood glucose level in the db/db + LYT mice decreased to the level almost equal to the level of control mice. Furthermore, the blood glucose level of the db/db + LYT group mice remained pretty low until 20 weeks old. In contrast, the db/db mice fed with LWE showed a greater reduction in blood glucose level during the first four weeks (12 to 16 weeks old) of treatment and then stayed at a high level (around 300 mg/dL) until 20 weeks old. These data suggest that the LYT dietary supplement had a better effect on reducing blood glucose levels than did the LWE dietary supplement in middle-age db/db mice.

The glucose tolerance test (GTT) showed no significant difference between db/db + SC and the db/db + LWE mice at 18 weeks old (Figure 3B). However, db/db + LYT mice efficiently catabolized the blood glucose, despite failing to restore near normal values after 120 min of glucose injection (Figure 3B). We found that LYT had a better effect on raising glucose tolerance than did LWE in db/db mice at the age of 18 weeks

### 2.5. LYT and LWE Attenuate Hepatic Steatosis in db/db Mice

**Figure 4:**
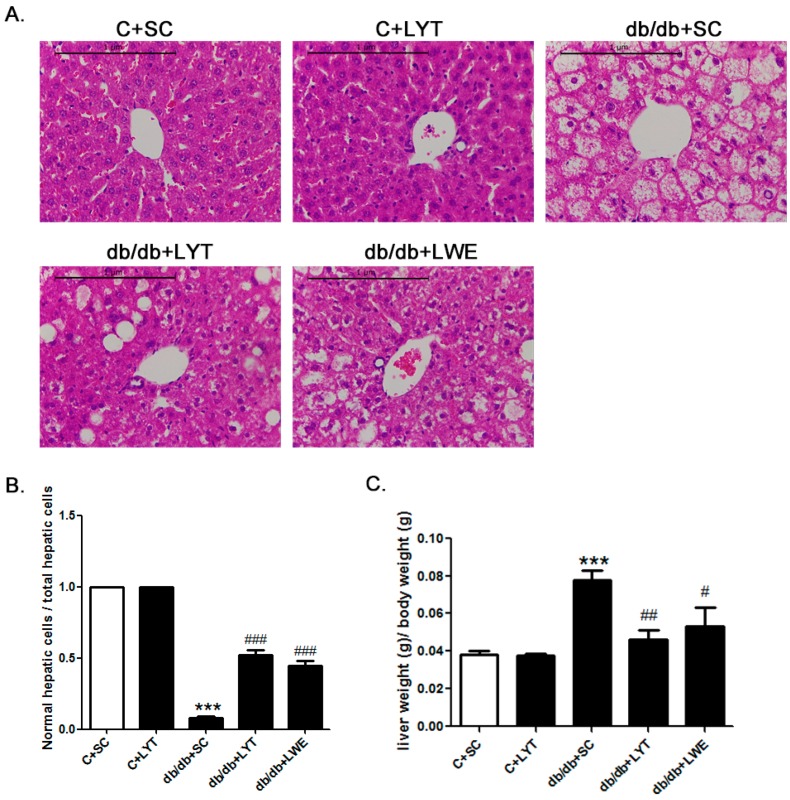
LYT and LWE diets attenuate hepatic steatosis and reduce liver-to-body weight ratio in db/db mice. Liver hematoxylin–eosin HE staining (**A**); normal hepatic cells to total liver cell ratio (5 field of view were randomly selected from each tissue sections for statistics (**B**); and liver-to-body weight ratio (**C**). Scale bars are equal to 1 μm. Values are means ± SE (*n* = 4–6). *** *p* < 0.001 when compared with the C + SC group; # *p* < 0.05; ## *p* < 0.01; ### *p* < 0.001 when compared with the db/db + SC group.

The liver weight to body weight ratio is an important index for fatty liver. Our data showed that the liver weight to body weight ratio in db/db mice fed a chow diet was significantly higher than that of the control mice fed a chow diet. Additionally, the db/db + LYT and db/db + LWE group mice showed a significantly decreased liver-to-body weight ratio when compared with that of the db/db mice fed a chow diet (Figure 4C). Furthermore, this index in the db/db + LYT mice was lower than that in the db/db + LWE mice, suggesting that LYT had a better effect on preventing fatty liver formation than LWE.

To better understand the protective effect of LYT and LWE on liver tissue structure in the five groups of mice, we examined the hepatic histology (Figure 4A). After 10 weeks of feeding with LYT, there was no difference in hepatic lobules between the C + SC and C + LYT mice. However, we found that the db/db mice with a standard chow diet had severe nonalcoholic fatty liver cells. Obviously, we observed diffuse hepatic fatty infiltration and amyloidosis in liver tissues, which caused a disruption in the structure of hepatic lobules in db/db mice fed a standard chow diet. In db/db mice fed with LYT and LWE diets, the liver tissues had smaller areas of fatty infiltration in liver cells, and the structure of hepatic lobules was integral. We also calculated the ratio of normal liver cells to total hepatic cells in the visual fields. We observed that db/db mice fed with LYT and LWE diets showed more normal liver cells than those of db/db mice fed a standard chow diet (Figure 4B). Again, the db/db mice fed with LYT showed more profoundly influenced the attenuation of hepatic steatosis when compared to the db/db mice fed with LWE.

### 2.6. LYT and LWE Reduced Lipid Accumulation in Liver Tissues via Suppressing the Lipogenesis in db/db Mice

**Figure 5:**
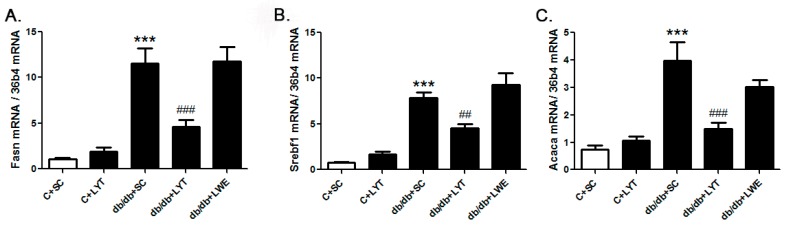
LYT diet decreases hepatic lipogenic gene expression in db/db mice. *Fasn* (**A**); *Sebf1* (**B**); and *Acaca* (**C**). Values are means ± SE (*n* = 4–6). *** *p* < 0.001 when compared with the C + SC group; ## *p* < 0.01; ### *p* < 0.001 when compared with the db/db + SC group.

Our data proved that LYT and LWE dietary supplements played a pivotal role in improving serum lipid profile and protecting fatty liver formation in db/db mice. To figure out the underlying mechanism, we measured the mRNA levels of genes related to the lipid metabolism in liver tissue. The hepatic gene expression data showed a significant increase in fatty acid synthase (*Fasn*), sterol regulatory element-binding transcription factor 1 (*Srebf1*), and acetyl-CoA carboxylase α(*Acaca*) in db/db mice with a standard chow diet when compared with control mice fed a standard chow diet (Figure 5A–C). *Acaca*, *Fasn*, and *Srebf1* are genes that work in conjunction to regulate lipogenesis [13,14,28,29]. However, the level of gene expression of *Acaca*, *Fasn*, and *Srebf1* was significantly decreased in db/db mice fed with LYT when compared with db/db mice fed a standard chow diet. In addition, no greater difference was observed in the level of these lipogenesis genes between the db/db mice fed with LWE and a standard chow diet (Figure 5A–C).

**Figure 6:**
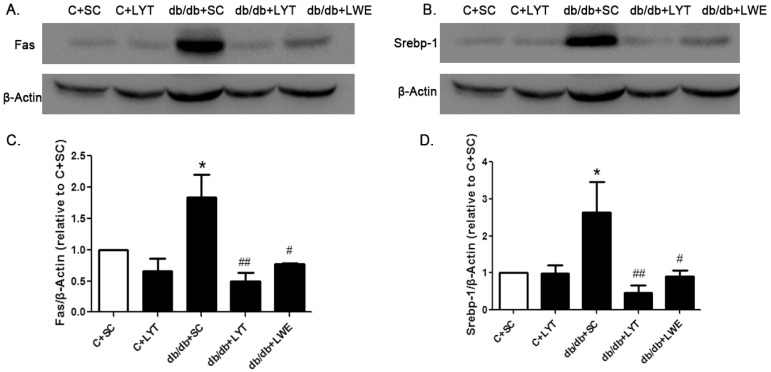
LYT and LWE diets decreased hepatic FAS and SREBP-1 protein expression in db/db mice. Representative figure (**A**) and summarized data (**C**) for FAS; representative figure (**B**) and summarized data (**D**) for SREBP-1. Values are means ± SE (*n* = 4–6). * *p* < 0.05 when compared with the C + SC group; # *p* < 0.05; ## *p* < 0.01 when compared with the db/db + SC group.

As the db/db mice supplemented with the LYE diet showed the significant downregulation of genes mediating lipogenesis, we further analyzed the protein expression levels of FAS and SREBP-1 between the five groups of mice. In the db/db mice fed a standard chow diet, the expression level of FAS and SREBP-1 was significantly higher than that in the control mice fed a standard chow diet, which meant that lipid synthesis in the liver was significantly accelerated in db/db mice when compared to control mice. However, the db/db mice fed LYT and LWE showed a significant decrease in the protein expression level of FAS and SREBP-1 in contrast to the db/db mice fed a standard chow diet (Figure 6A–D). Therefore, our data suggested that the effects of LYT and LWE on preventing fatty liver formation and improving serum lipid profile in db/db mice after 10 weeks of dietary supplement might be due to the suppression of hepatic lipogenesis.

**Figure 7:**
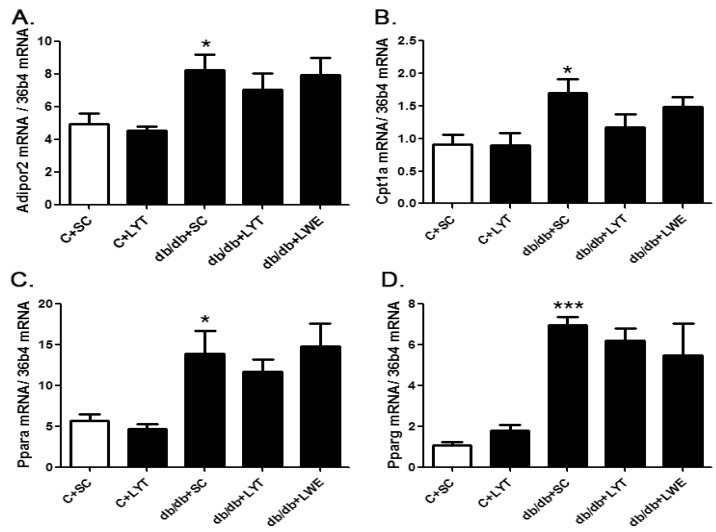
The mRNA expression of genes involved in lipolysis in the liver of control and db/db mice at age of 20 weeks. *Adipor2* (**A**); *Cpt1a* (**B**); *Pparα*(**C**); and *Pparγ* (**D**). Values are means ± SE (*n* = 4–6). * *p* < 0.05; *** *p* < 0.001 when compared with the C + SC group.

Interestingly, we found that there was no upregulation of predominant lipolysis genes such as the adiponectin receptor protein 2 (*Adipor2*), carnitine palmitoyltransferase 1A (*Cpt1a*), peroxisome proliferator-activated receptor alpha (*Pparα*), and peroxisome proliferator-activated receptor gamma *(Pparγ*) in the livers of db/db mice fed with LYT and LWE when compared to the db/db mice fed a standard chow diet (Figure 7A–D).

### 2.7. LYT Improved the Hepatic Glycometabolism in db/db Mice

**Figure 8:**
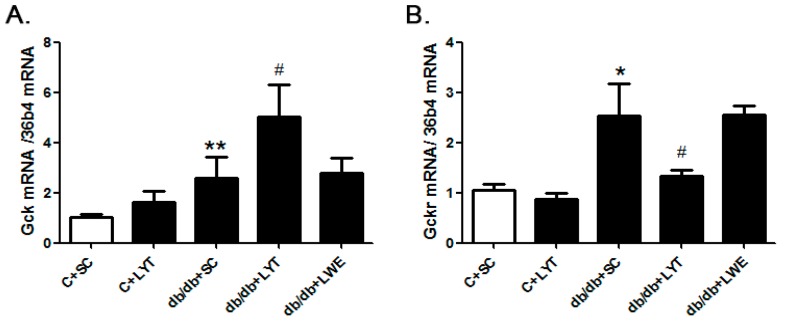
The mRNA expression of *Gck* (**A**) and *Gckr* (**B**) in the liver of the control and db/db mice at 20 weeks old. Values are means ± SE (*n* = 4–6). * *p* < 0.05; ** *p* < 0.01 when compared with the C + SC group; # *p* < 0.05 when compared with the db/db + SC group.

The hepatic glucokinase (GCK) and glucokinase regulatory protein (GCKR) play key roles in glucose homeostasis by increasing or decreasing glucose output and uptake during fasting and feeding. To identify the mechanism of LYT diets decreasing blood glucose levels in db/db mice, we examined the mRNA expressions of *Gsk* and *Gskr* in the liver tissues. The real-time polymerase chain reaction (PCR) data revealed that the expression level of *Gck* in the db/db + SC mice liver was significantly increased when compared with that of the control mice. Moreover, the *Gck* expression level in the liver was further increased in the db/db + LYT mice when compared with the db/db + SC mice (Figure 8A). GCKR controls both the activity and intracellular location of glucokinase through binding and moving this key enzyme of glycometabolism [30,31,32,33]. Therefore, we also measured the expression of *Gckr* in the liver among the five groups of mice. Our data showed that the db/db + SC mice showed an increased level of *Gckr* mRNA expression in contrast to the control mice, and *Gckr* expression showed a significant reduction in db/db mice fed LYT when compared to the db/db mice fed a standard chow diet (Figure 8B). However, the db/db mice fed LWE saw no increase in *Gck* or any decrease in *Gckr* expression when compared to the db/db mice fed a standard chow diet. These results potentially validated the antagonism between *Gck* and *Gckr.*

## 3. Discussion

It is demonstrated for the first time here that large yellow tea is a potential natural health product that attenuates nonalcoholic fatty liver and symptoms of T2D in db/db mice including hyperlipidemia and hyperglycemia. Quantitative analysis of the total catechins provided a record of ECGC having the highest level of presence in both LYT and LWE than other catechins subjected to quantification. As many previous studies have investigated the health benefits of pure EGCG, we designed our experiment to keep almost the same concentration of EGCG in both the LYT and LWE diet. If there were any deferent phenotypes between the db/db + LYT and db/db + LWE group mice, we could speculate that the phenotypes were effects of the other catechins or even other functional components rather than EGCG. Considering the fact that people consume whole tea as a beverage or food supplements, it was important to investigate the health benefits of tea powder and water extract. In this experiment, a 5% LYT diet was used to feed mice, corresponding to the 20 g daily tea consumption of human [22]. Twenty grams of tea is approximately equal to drinking five cups of tea. This is a reasonable daily amount for regular tea consumers.

It has been reported that teas and their extracts, especially green tea and black tea, have an effect on losing weight in diet-induced obesity and diabetic rodent models [34,35], which was further confirmed in human populations [36,37]. However, the beneficial effect of large yellow tea on losing weight in db/db mice has yet to be reported. db/db mice are characterized by polydipsia, polyphagia, obesity, and weight loss at later stages concerned with carbohydrate and lipid metabolic disorder [13,38]. In our research, the db/db mice showed the phenotype characteristic of excessive eating and drinking as well as obesity, which was not significantly recorded in control mice since the age of 10 weeks (Figure 1A–G). However, the db/db mice fed LYT and LWE showed significantly decreased food consumption and water intake during the entire experimental period. These data suggest that dietary supplements of LYT and LWE efficiently improved diabetic syndrome of db/db mice. The db/db + SC group of mice gradually gained weight until 14 weeks and then steadily lost weight from 14 weeks to the end of the experiment at 20 weeks due to the development of the T2D complication. However, the db/db mice fed with LYT and LWE showed a consistent increase in body weight until the age of 16 weeks (Figure 1G), then gradually lost weight at a degree less than the db/db + SC group mice. Our results indicated that LYT and LWE diets could delay the development of diabetic complications in db/db mice.

The liver is a key metabolic organ that governs glucose and lipid metabolism and plays important roles in the balance of energy [39,40]. Dysregulation of lipid metabolism leads to hepatic damage, and even causes severe liver diseases [41]. Hyperglycemia and hyperlipidemia are important phenotypes of metabolic syndrome in db/db mice [13,38]. It has been reported that green tea as well as black tea decreased lipid accumulation via increasing lipolysis and suppressing lipogenesis in the liver of T2D rodent models [34,35]. In our research, the mRNA expression levels of *Acaca*, *Fasn* and *Srebf1* in liver tissues were significantly increased in db/db mice fed a standard chow diet when compared with the control mice. *Acaca*, *Fasn*, and *Srebf1* were all involved in lipid synthesis, which indicated increased lipogenesis in the db/db + SC group of mice (Figure 5A–C). However, the mRNA expression levels of *Acaca*, *Fasn*, and *Srebf1* in the livers of db/db mice treated with LYT were significantly attenuated when compared to db/db mice fed a standard chow diet at 20 weeks old. In addition, Western blot results showed that the protein expression levels of FAS and SREBP1 were also dramatically decreased in the liver tissues of db/db mice fed with LYT and LWE when compared to that of db/db mice fed a standard chow diet. Additionally, FAS or SREBP1 is a key enzyme or a transcriptional factor in regulating lipid and glucose metabolism. These results demonstrated that LYT and LWE dietary supplements regulated the glucose and lipid metabolism in db/db mice by decreasing the FAS and SREBP1 expression. Furthermore, this set of data was consistent with the decreased levels of TG, LDL-C, and TC in the serum of db/db mice fed with LYT (Figure 2A–C). In contrast, our data indicated that the mRNA levels of main lipolysis genes, *Adipor2*, *Cpt1a*, *Pparα*, and *Pparγ* were not altered in the liver of db/db mice fed with LYT and LWE when compared to db/db mice fed a standard chow diet (Figure 7A–D). Put together, our data suggested that dietary supplements of LYT or LWE decreased lipid accumulation and that the prevention of fatty liver formation might be involved in suppressing lipogenesis rather than increasing lipolysis. It is possible that the downregulation of *Srebf1* and its target gene *Fasn* catalyzes the downregulation of *Acaca* in fatty acid synthesis and that this potential molecule contributes to the reduction in de novo lipogenesis [42].

At the age of 10 weeks, the db/db + SC mice showed a significant increase of blood glucose level when compared to the C + SC mice (*p* < 0.001). Surprisingly, the blood glucose level was significantly decreased to a level very close to normal glucose levels in db/db mice fed with LYT from 14 to 20 weeks old (Figure 3A). In addition, db/db mice fed with LYT significantly raised glucose tolerance at the age of 20 weeks (Figure 3B). This result is very important as the dietary supplement of LYT had a very strong effect on decreasing blood glucose in the T2D db/db mice, while the dietary supplement of LWE significantly decreased the blood glucose level of db/db mice for only the first two weeks of treatment (12–14 weeks old), and blood glucose then gradually increased from 14 to 16 weeks and stayed at approximately 300 mg/dL until the end of the experiment (20 weeks). What we found here was that a dietary supplement of large yellow tea powder was better able to decrease blood glucose than the water extract supplement in db/db mice.

We further investigated the mechanism of this effect. GCKR, a protein highly expressed in liver cells, plays a crucial role in the inhibition of GCK activity and thus accordingly regulates glucose metabolism in T2D rodent models. Previous studies have reported that GCK activity is decreased in T2D [30,31,32,33]. At the age of 20 weeks, we found that the mRNA expression level of *Gckr* in db/db mice was higher than that in the control mice (Figure 8B), which suggested that db/db mice developed a glucose metabolism disorder by increasing the expression of GCKR and further decreasing the activity of GCK. This result is consistent with the results reported previously [43,44,45]. As expected, when compared with the db/db mice fed a standard chow diet, the mRNA expression level of *Gckr* in the liver of db/db mice fed the LYT diet was significantly decreased. Accordingly, the mRNA expression level of *Gck* showed obvious upregulation in the db/db mice fed LYT (Figure 8A). These data suggested that the dietary supplement of large yellow tea powder may regulate glucose metabolism in T2D db/db mice by accordingly decreasing the expression of GCKR and increasing the expression of GCK in the liver. However, there were no significant differences in the mRNA expressions of *Gckr* and *Gck* between the db/db + SC mice and db/db + LWE mice at the age of 20 weeks. This may be a reason why the water extract of large yellow tea had less effect on reducing blood glucose levels and improving glucose tolerance in db/db mice.

Overweight, obesity, and diabetes are emerging as major global health issues. If tea could prevent or delay the development of these diseases, the public health implications would be tremendous [17]. So far, most of the beneficial effects of tea are believed to be attributed to the polyphenols in the tea. Although there have been many published studies, the exact functional components and molecular mechanisms are still not fully understood. Yellow tea, as well as green tea and white tea, contain characteristic polyphenolic compounds known as catechins, and EGCG is the major form of tea catechin [46]. Therefore, previous research has mainly focused on EGCG inhibiting obesity, metabolic syndrome in db/db mice [21], and high-fat-diet-fed mice [46,47,48]. Similar results were also observed using black tea extract (BTE) [34] and green tea extract (GTE) [49]. To the best of our knowledge, this is the first work to comparatively investigate the effect of dietary supplements of large yellow tea powder and its water extract on metabolic syndrome, serum lipid profile, and hepatic steatosis in db/db mice. Most of the data generated by this study have shown that dietary supplement of large yellow tea powder has more profound protective effects than its water extract in metabolic syndrome, especially in regulating glucose metabolism in the middle stage of T2D in db/db mice. As stated previously, we designed our study to keep almost the same concentration of EGCG in the large yellow tea powder diet and its water extract diet. Our results suggested that the other catechins or even other chemical components rather than EGCG may play important roles in alleviating metabolic syndrome, especially in regulating glucose metabolism in db/db mice. The water extract of large yellow tea contains only water-soluble components, while large yellow tea contains not only water-soluble substances, but also various fat-soluble substances [16,17]. Therefore, we hypothesize that a range of substances not extracted by water in large yellow tea may contribute the principal effects of decreasing blood glucose and alleviating metabolic syndrome in db/db mice. These exact functional components need further investigation.

## 4. Materials and Methods

### 4.1. The Extract of Large Yellow Tea

The large yellow tea purchased from Bao Er Zhong Xiu Tea Industry Co., Ltd. (Huoshan, Anhui, China) was extracted with pure water at 100 °C (the ratio of tea powder to pure water was 1:20), for 20 min, then extraction was assisted by ultrasonic (KQ-500DE Shumei, Kunshan, China) in a water bath for 30 min at 75 °C. The resulting water extract of large yellow tea was further filtered and subjected to concentration using a rotary evaporator (IKA^®^ HB 10, Staufen, German). Finally, the concentrated extraction was freeze-dried to form a solid powder in order to add to the AIN93 standard diet for experiment.

### 4.2. Analysis of Major Chemical Components of Large Yellow Tea and Its Water Extract

The catechins, theanine, and caffeine were analyzed on a Waters High Performance Liquid Chromatography (HPLC) system supported with a Waters 600 controller and Waters 2489 UV/Visible Detector (280 nm). Chromatographic separation was performed on a Phenomenex Gemini C18 column. The column temperature was set at 25 °C. The injection volume of sample was 5 μL, the elution rate was 1 mL/min, and the detection wavelength was set at 278 nm. The mobile phase consisted of mobile phase A (deionized water with 0.17% acetic acid) and mobile phase B (100% acetonitrile). The linear gradient at a flow rate of 1.0 mL/min was set as follows: mobile phase B from 8–28.4% (*v*/*v*) in 30 min was initiated, from 28.4–100% (*v*/*v*) for 8 min, and from 100–8% (*v*/*v*) for another 10 min.

### 4.3. Animal Experiments

C57BLKsJ-db/ + mice (control mice, *n* = 12) and male C57BLKsJ-db/db (db/db mice, *n* = 18) were purchased from the National Resource Center of Model Mice (NRCMM, Nanjing, China) and allowed to acclimate to the environment of the specific pathogen free (SPF) laboratory animal center at Anhui Agricultural University, which was controlled with a constant temperature (22 ± 1 °C) and humidity (50 ± 5%) under a 12:12 h light–dark cycle falls on 8:00 a.m. to 8:00 p.m. All animals were housed in cages and had free access to water and diet. Animals were fed normally until 10 weeks old, and then control mice were randomly divided into two groups, while db/db mice were divided into three groups fed with different diets. The detailed group assignments for the mice are described in Figure 1. All animal procedures were approved by the Institutional Animal Care and Use Committee of the Anhui Agricultural University (ethical approval code: AHAU 2016-007).

The standard AIN93 diet (SC), SC containing 5% of the large yellow tea powder (LYT), and SC containing 1.5% of the large yellow tea water extract (LWE) were purchased from Trophic Animal Feed High-Tech Co., Ltd. (Nantong, China). The water consumption and food intake were monitored daily, fasting blood glucose level, and body weights were measured at weekly intervals. Blood was obtained from the tail, and the glucose concentrations were measured using Nova StatStrip Xpresst^M^ Glucose CR Meter (Nova Biomedical, Waltham, UK) with Nova StatStrip Xpresst^M^ Glu-test Strips (Nova Biomedical, Waltham, UK).

At the end of the 10 week dietary treatments, the mice fasted for 12 h, were anesthetized via injection with 4% chloral hydrate (10 mL/kg, i.p.), and were then sacrificed after the peripheral blood collection from the ophthalmic vein. Serum was obtained by centrifugation at 3000 rpm/min for 5 min at 4 °C then stored at −80 °C. The levels of triglyceride (TG), low-density lipoprotein cholesterol (LDL-C), high-density lipoprotein cholesterol (HDL-C), and total cholesterol (TC) in the serum were measured using micro test kits from Nanjing Jiancheng Bioengineering Institute (Nanjing, Jiangsu, China). Liver weights were measured on a scale to determine liver mass. All other tissue samples were harvested for further biochemical, molecular, and immunostaining analyses. Small pieces of the liver tissues were preserved in RNAlater solution for gene expression experiments, and the other small pieces of the liver tissues were fixed in formaldehyde solution from ZHANYUN (Wuxi, Jiangsu, China) at 4 °C preserved for histological experiment, and the rest of the liver tissues were immediately liquid nitrogen frozen before being stored at −80 °C for the protein expression experiment.

### 4.4. Glucose Tolerance Test

Glucose tolerance test (GTT) were performed at the age of 18 weeks. The fasting mice were given an intraperitoneal injection of glucose (d-(+)-Glucose (SIGMA, St. Louis, MO, USA) at the dosage of 1.5 g/kg body weight and the blood samples were collected from tail veins of the mice and glucose levels were measured at 0, 30, 60, 90, and 120 min before and after injection [50].

### 4.5. Hematoxylin–Eosin Staining

All of the liver tissues for the histological chemistry experiment were fixed in 10% neutrally buffered formalin solution at room temperature, dehydrated, and embedded in paraffin (Paraplast Tissue Embedding Medium, LEICA, Buffalo Grove, IL, USA) using a modular tissue embedding system (LEICA EG1150 H, Buffalo Grove, IL, USA). Hematoxylin–eosin (HE) staining was carried out on 5 μm sections using a fully automated rotary microtome (LEICA RM2255, Nussloch, Germany) and mounted onto positive charged slides (Adhesion Microscope Slides, CITOGLAS, Shanghai, China). HE staining of the liver paraffin sections was performed using an HE staining kit (Boster Biological Technology Company, Pleasanton, California, USA) and was pictured by microscope (LEICA DM500, Wetzlar, Germany) with a supporting camera (LEICA ICC50 W, Wetzlar, Germany). The hepatic adipose infiltration cells were counted manually using ImageJ software (Version 1.51q).

### 4.6. Real-Time Polymerase Chain Reaction PCR

Total RNA was extracted from the liver tissues, which were fixed in RNA stabilization solution (Thermo Fisher Scientific, Waltham, Massachusetts, USA) at −80 °C, and was isolated using RNA isolator (Vazyme Biotech Co., Ltd., Nanjing, Jiangsu, China) as per the manufacturer’s instructions. Reverse transcription was performed using HiScript^®^ II 1st Strand cDNA Synthesis kit (Vazyme Biotech Co., Ltd., Nanjing, China), and reverse transcriotion-polymerase chain reaction (RT-PCR) was performed using the Bio-Rad CFX System and AceQ qPCR SYBR Green Master Mix kit (Vazyme Biotech Co., Ltd., Nanjing, China). Real-time PCR was performed following the method described previously in [51,52,53,54]. Primer sequences were designed for mice and listed in Table 2.

### 4.7. Western Blot Analysis

Western blot was performed following the method described previously in [52,53,54]. In brief, the frozen liver tissues were homogenized using a 2 × SDS buffer. Equal amounts of denatured proteins were separated by SDS-PAGE gels and transferred to nitrocellulose membranes. The membranes were blocked with 5% skimmed milk powder in PBS-T solution for 1 h and were incubated with fatty acid synthase (FAS) (Santa Cruz Biotechnology, Santa Cruz, CA, USA), sterol regulatory element-binding protein-1 (SREBP-1) (Santa Cruz Biotechnology, Santa Cruz, CA, USA), and β-Actin (Proteintech^TM^, Wuhan, Hubei, China) at 4 °C overnight, then incubated with appropriate secondary antibodies (Proteintech^TM^, Wuhan, Hubei, China) for 1 h at room temperature. Protein bands were detected by enhanced chemiluminescent (ECL) reagent (Vazyme Biotech Co., Ltd., Nanjing, China) and analyzed using the ChemicDoc^TM^ MP Imaging System (Bio-Rad, Hercules, CA, USA) with supporting system (ImageLab., Bio-Rad, Hercules, CA, USA).

### 4.8. Statistical Analysis

The results were expressed as mean ± SEM. Comparisons between the two groups were performed with an unpaired 2-tailed Student’s *t*-test, and multiple group comparisons were performed by one-way ANOVA followed by Tukey’s test. *p* < 0.05 was used to consider statistical significance.

## Figures and Tables

**Table 1:** Quantitative data for catechins, caffeine, and theanine in large yellow tea (LYT) and its water extract (LWE) via HPLC analysis.

	Caffeine	EGC	C	EC	EGCG	GCG	ECG	Theanine
LWE (mg/g)	18.766 ± 0.394	1.133 ± 0.464	0.536 ± 0.096	1.231 ± 0.045	13.774 ± 0.543	2.970 ± 0.107	4.117 ± 0.145	7.625 ± 1.257
LYT (mg/g)	8.906 ± 0.282 **	0.731 ± 0.003 ***	0.184 ± 0.002 *	0.811 ± 0.002 ***	4.250 ± 0.023 ***	0.877 ± 0.002 ***	1.237 ± 0.004 ***	0.648 ± 0.166 ***

Notes: Values are means ± SE (*n* = 3). * *p* < 0.05, ** *p* < 0.01, *** *p* < 0.001 when compared with large yellow tea water extract (LWE)*.*

**Table 2:** Primer sequences used for RT-PCR gene expression experiment.

	Primer Sequences	
Gene	Forward (5′–3′)	Reverse (5′–3′)
*36b4*	CCC TGA AGT GCT CGA CAT CA	TGC GGA CAC CCT CCA GAA
*Fasn*	CGT GTG ACC GCC ATC TAT ATC G	TGA GGT TGC TGT CGT CTG TAG TCT T
*Srebf1*	AGT CCA GCC TTT GAG GAT AGC C	CCG TAG CAT CAG AGG GAG TGA G
*Acaca*	AGG AGG GAA AGG GAT CAG AAA AG	CAG AGC AGT CAC GAC CAA ACA AA
*Adipor2*	CCT TTC GGG CCT GTT TTA AGA	GAG TGG CAG TAC ACC GTG TG
*Cpt1a*	CTT CAA AAA CAG CAA GAT AGG CAT A	TTA CAG TGT CCA TCC TCT GAG TAG C
*Ppara*	TAC GCT CCC GAC CCA TCT TTA	GAC TCC TTG GCA GTG TCC ATC T
*Pparg*	GAA AGA CAA CGG ACA AAT CAC CAT	CGG CTT CTA CGG ATC GAA ACT G
*Gck*	AGA CGA AAC ACC AGA TGT ATT CC	GAA GCC CTT GGT CCA GTT GAG
*Gckr*	GAC CCG GAA CTT GGA CAA AG	AAT GCC ATA CGA CCA GAG GTG

## References

[1] Dandona P., Aljada A., Chaudhuri A., Mohanty P., Garg R. (2005). Metabolic syndrome, a comprehensive perspective based on interactions between obesity, diabetes, and inflammation. Circulation.

[2] Forbes J.M., Fotheringham A.K. (2017). Vascular complications in diabetes: Old messages, new thoughts. Diabetologia.

[3] Alberti K.G., Zimmet P., Shaw J. (2005). The metabolic syndrome-a new worldwide definition. Lancet.

[4] Schulman R.C., Mechanick J.L. (2012). Metabolic and nutrition support in the chronic cirtical illness syndrome. Respir. Care.

[5] Wilson P.W., D’Agostino R.B., Parise H., Sullivan L., Meigs J.B. (2005). Metabolic syndrome as a precursor of cardiovascular disease and type 2 diabetes mellitus. Circulation.

[6] Oda E. (2012). Metabolic syndrome: Its history, mechanisms, and limitations. Acta Diabetol..

[7] Cryer M.J., Horani T., DiPette D.J. (2016). Diabetes and Hypertension: A Comparative Review of Current Guidelines. J. Clin. Hypertens..

[8] Vague J. (1956). The degree of masculine differentiation of obesities: A factor determining predisposition to diabetes, atherosclerosis, gout, and uric calculous disease. Am. J. Clin. Nutr..

[9] Reaven G.M. (1993). Role of insulin resistance in human disease (syndrome X): An expanded definition. Annu. Rev. Med..

[10] Samson S.L., Garber A.J. (2014). Metabolic syndrome. Endocrinol. Metab. Clin. N. Am..

[11] Engin A. (2017). The Definition and Prevalence of Obesity and Metabolic Syndrome. Adv. Exp. Med. Biol..

[12] Sandouk Z., Lansang M.C. (2017). Diabetes with obesity—Is there an ideal diet?. Clevel. Clin. J. Med..

[13] Su W., Guo Z., Randall D.C., Cassis L., Brown D.R., Gong M.C. (2008). Hypertension and disrupted blood pressure circadian rhythm in type 2 diabetic db/db mice. Am. J. Physiol. Heart Circ. Physiol..

[14] Lee Y., Berglund E.D., Yu X., Wang M.Y., Evans M.R., Scherer P.E., Holland W.L., Charron M.J., Roth M.G., Unger R.H. (2014). Hyperglycemia in rodent models of type 2 diabetes requires insulin-resistant alpha cells. Proc. Natl. Acad. Sci. USA.

[15] Sharma K., McCue P., Dunn S.R. (2003). Diabetic kidney disease in the db/db mouse. Am. J. Physiol. Ren. Physiol..

[16] Ning J., Li D., Luo X., Ding D., Song Y., Zhang Z., Wan X. (2016). Stepwise identification of six tea (*Camellia sinensis* (L.) categories based on catechins, caffeine, and theanine contents combined with fisher discriminant analysis. Food Anal. Method.

[17] Yang C.S., Zhang J., Zhang L., Huang J., Wang Y. (2016). Mechanisms of body weight reduction and metabolic syndrome alleviation by tea. Mol. Nutr. Food Res..

[18] Bogdanski P., Suliburska J., Szulinska M., Stepien M., Pupek-Musialik D., Jablecka A. (2012). Green tea extract reduces blood pressure, inflammatory biomarkers, and oxidative stress and improves parameters associated with insulin resistance in obese, hypertensive patients. Nutr. Res..

[19] Paradee A., Montira P., Oratai T., Bung-orn S., Narong A., Bandit T., Soontorn K., Srisuda W., Supat S., Pranithi H. (2008). Effectiveness of green tea on weight reduction in obese Thais: A randomized, controlled trail. Physiol. Behav..

[20] Kang S.J., Lee J.E., Lee E.K., Jung D.H., Song C.H., Park S.J., Choi S.H., Han C.H., Ku S.K., Lee Y.J. (2014). Fermentation with Aquilariae Lignum enhances the anti-diabetic activity of green tea in type II diabetic db/db mouse. Nutrients.

[21] Ortsäter H., Grankvist N., Wolfram S., Kuehn N., Sjöholm A. (2012). Diet supplementation with green tea extract epigallocatechin gallate prevents progression to glucose intolerance in db/db mice. Nutr. Metab..

[22] Han M., Zhao G., Wang Y., Wang D., Sun F., Ning J., Wan X., Zhang J. (2016). Safety and anti-hyperglycemic efficacy of various tea types in mice. Sci. Rep..

[23] Stuart E.C., Jarvis R.M., Rosengren R.J. (2010). In vitro mechanism of action for the cytotoxicity elicited by the combination of epigallocatechingallate and raloxifene in MDA-MB-231 cells. Oncol. Rep..

[24] Isbrucker R.A., Edwards J.A., Wolz E., Davidovich A., Bausch J. (2006). Safety studies on epigallocatechingallate (EGCG) preparations. Part 2: Dermal, acute and short-term toxicity studies. Food Chem. Toxicol..

[25] Kucera O., Mezera V., Moravcova A., Endlicher R., Lotkova H., Drahota Z., Cervinkova Z. (2015). In vitro toxicity of epigallocatechingallate in rat liver mitochondria and hepatocytes. Oxid. Med. Cell. Longev..

[26] Kim K.E., Jung Y., Min S., Nam M., Heo R.W., Jeon B.T., Song D.H., Yi C.O., Jeong E.A., Kim H. (2016). Caloric restriction of db/db mice reverts hepatic steatosis and body weight with divergent hepatic metabolism. Sci. Rep..

[27] Porcellati F., Lucidi P., Bolli G.B., Fanelli C.G. (2013). Thirty years of research on the dawn phenomenon: Lessons to optimize blood glucose control in diabetes. Diabetes Care.

[28] Fullerton M.D., Galic S., Marcinko K., Sikkema S., Pulinilkunnil T., Chen Z.P., O’Neill H.M., Ford R.J., Palanivel R., O’Brien M. (2013). Single phosphorylation sites in Acc1 and Acc2 regulate lipid homeostasis and the insulin-sensitizing effects of metformin. Nat. Med..

[29] Geng F., Cheng X., Wu X., Yoo J.Y., Cheng C., Guo J.Y., Mo X., Ru P., Hurwitz B., Kim S.H. (2016). Inhibition of SOAT1 suppresses glioblastoma growth via blocking SREBP-1-mediated lipogenesis. Clin. Cancer Res..

[30] Hattersley A.T., Tooke J.E. (1999). The fetal insulin hypothesis: An alternative explanation of the association of low birth weight with diabetes and vascular disease. Lancet.

[31] Callejas D., Mann C.J., Ayuso E., Lage R., Grifoll I., Roca C., Andaluz A., Ruiz-de G.R., Montané J., Muñoz S. (2013). Treatment of diabetes and long-term survival after insulin and glucokinase gene therapy. Diabetes.

[32] Zelent B., Raimondo A., Barett A., Buettger C.W., Chen P., Gloyn A.L., Matschinsky F.M. (2014). Analysis of the co-operative interaction between the allosterically regulated proteins GK and GKRP using tryptophan fluorescence. Biochem. J..

[33] Lloyd D.J., St Jean D.J., Kurzeja R.J., Wahl R.C., Michelsen K., Cupples R., Chen M., Wu J., Sivits G., Helmering J. (2013). Antidiabetic effects of glucokinase regulatory protein small-molecule disruptors. Nature.

[34] Uchiyama S., Taniguchi Y., Saka A., Yoshida A., Yajima H. (2011). Prevention of diet-induced obesity by dietary black tea polyphenols extract in vitro and in vivo. Nutrition.

[35] Bruno R.S., Dugan C.E., Smyth J.A., DiNatale D.A., Koo S.I. (2008). Green tea extract protects leptin-deficient, spontaneously obese mice from hepatic steatosis and injury. J. Nutr..

[36] Jurgens T.M., Whelan A.M., Killian L., Doucette S., Kirk S., Foy E. (2012). Green tea for weight loss and weight maintenance in overweight or obese adults. Cochrane Database Syst. Rev..

[37] Hursel R., Viechtbauer W., Westerterp-Plantenga M.S. (2009). The effects of green tea on weight loss and weight maintenance: A meta-analysis. Int. J. Obes..

[38] Zhao C., Qiao C., Tang R.H., Jiang J., Li J., Martin C.B., Bulaklak K., Li J., Wang D.W., Xiao X. (2015). Overcoming insulin insuffciency by forced follistatin expression in β-cells of db/db mice. Mol. Ther..

[39] Bechmann L.P., Hannivoort R.A., Gerken G., Hotamisligil G.S., Trauner M., Canbay A. (2012). The interaction of hepatic lipid and glucose metabolism in liver diseases. J. Hepatol..

[40] Marchesini G., Petta S., Dalle G.R. (2016). Diet, weight loss, and liver health in nonalcoholic fatty liver disease: Pathophysiology, evidence, and practice. Hepatology.

[41] Lee S., Mardinoglu A., Zhang C., Lee D., Nielsen J. (2016). Dysregulated signaling hubs of liver lipid metabolism reveal hepatocellular carcinoma pathogenesis. Nucleic Acids Res..

[42] Ameer F., Scandiuzzi L., Hasnian S., Kalbacher H., Zaidi N. (2014). De novo lipogenesis in health and disease. Metabolism.

[43] Bonetti S., Trombetta M., Boselli M.L., Turrini F., Malerba G., Trabetti E., Pignatti P.F., Bonora E., Bonadonna R.C. (2011). Variants of GCKR affect both β-cell and kidney function in patients with newly diagnosed type 2 diabetes: The Verona newly diagnosed type 2 diabetes study 2. Diabetes Care.

[44] Hu C., Zhang R., Wang C., Yu W., Lu J., Ma X., Wang J., Jiang F., Tang S., Bao Y., Xiang K., Jia W. (2010). Effects of GCK, GCKR, G6PC2 and MTNR1B variants on glucose metabolism and insulin secretion. PLoS ONE.

[45] Haeusler R.A., Camastra S., Astiarraga B., Nannipieri M., Anselmino M., Ferrannini E. (2014). Decreased expression of hepatic glucokinase in type 2 diabetes. Mol. Metab..

[46] Bose M., Lambert J.D., Ju J., Reuhl K.R., Shapses S.A., Yang C.S. (2008). The major green tea polyphenol, (−)-epigallocatechin-3-gallate, inhibits obesity, metabolic syndrome, and fatty liver disease in high-fat–fed mice. J. Nutr..

[47] Chen Y.K., Cheung C., Reuhl K.R., Liu A.B., Lee M.J., Lu Y.P., Yang C.S. (2011). Effects of green tea polyphenol (−)-epigallocatechin-3-gallate on newly developed high-fat/Western-style diet-induced obesity and metabolic syndrome in mice. J. Agric. Food Chem..

[48] Byu J.K., Yoon B.Y., Jhun J.Y., Oh H.J., Kim E.K., Min J.K., Cho M.L. (2014). Epigallocatechin-3-gallate ameliorates both obesity and autoinflammatory arthritis aggravated by obesity by altering the balance among CD4+ T-cell subsets. Immunol. Lett..

[49] Okuda M.H., Zemdegs J.C., de Santana A.A., Santamarina A.B., Moreno M.F., Hachui A.C., dos Santos B., do Nascimento C.M., Ribero E.B., Oyama L.M. (2014). Green tea extract improves high fat diet-induced hypothalamic inflammation, without affecting the serotoninergic system. J. Nutr. Biochem..

[50] Tian L., Zeng K., Shao W., Yang B.B., Fantus I.G., Weng J., Jin T. (2015). Short-Term Curcumin Gavage Sensitizes Insulin Signaling in Dexamethasone-Treated C57BL/6 Mice. J. Nutr..

[51] Xie Z., Gong M.C., Su W., Turk J., Guo Z. (2007). Group VIA phospholipase A_2_ (iPLA_2_β) participates in angiotensin II-induced transcriptional up-regulation of regulator of G-protein signaling-2 in vascular smooth muscle cells. J. Biol. Chem..

[52] Xie Z., Su W., Guo Z., Pang H., Post S.R., Gong M.C. (2006). Up-regulation of CPI-17 phosphorylation in diabetic vasculature and high glucose cultured vascular smooth muscle cells. Cardiovasc. Res..

[53] Xie Z., Liu D., Liu S., Calderon L., Zhao G., Turk J., Guo Z. (2011). Identification of a cAMP-response element in the regulator of G-protein signaling-2 (RGS2) promoter as a key Cis-regulatory element for RGS2 transcriptional regulation by angiontensin II in cultured vascular smooth muscles. J. Biol. Chem..

[54] Xie Z., Su W., Liu S., Zhao G., Esser K., Schroder E.A., Lefta M., Stauss H.M., Guo Z., Gong M.C. (2015). Smooth-muscle BMAL1 participates in blood pressure circadian rhythm regulation. J. Clin. Investig..

